# The Value of Prophylactic Treatment in Anthrax

**Published:** 1894-12

**Authors:** John Faust

**Affiliations:** Poughkeepsie, N. Y.


### THE VALUE OF PROPHYLACTIC TREATMENT IN ANTHRAX.

By John Faust, V. S., Poughkeepsie, N. Y.

For twenty-nine years, to my personal knowledge, splenic apoplexy and splenic fever have occurred every year up to 1886, in a streak of swampy land about two miles long, and Mr. Noxon, one of the sufferers, says that he lost $30,000 worth of cattle in that period. All imaginable diseases were suspected by professional and non-professional gentlemen. I held post-mortems year after year, and always found one and the same thing—splenic fever, or apoplexy.

In the year 1885, the suspicion of poisoning was so strong that Mr. Nixon wanted a chemical analysis made of the stomach. As I knew that would have been money spent for nothing, I proposed that Professor Liantard make a post-mortem examination. After the post-mortem he stated to the owner that the whole cause was splenic fever.

A great many medicines were used with no effect. I advocated to the owners vaccination with anthrax virus. I even went so far as to offer to do it for nothing if it was a failure.

The following is a history of the different herds that came under my care in 1886:

On July 3d was called to a cow belonging to Mr. Hoppe She died before I reached her. The second cow was found dead in the lot. On July 10th was called to the third cow, and put her under treatment for splenic fever (my record does not show what treatment). In twelve hours to all appearances she was well, but in ten hours more she died, and post mortem examination proved it to be splenic fever. The spleen weighed eight and one-half pounds.

Mr. Hoppe's neighbor also lost his only cow. They were the only cows these men owned.

On July 17th I was called to Mr. E. Lyon's farm. Ten days previous to my call he lost one Jersey cow. On July 16th the second cow was taken sick, to which Dr. Thompson was called, and diagnosed it as dysentery. The cow died shortly after he left. On July 17th two more cows died without warning or medical care. The same day I found nine sick cows. My diagnosis was splenic fever. I was not prepared for any treatment that night. On the 18th I made a post-mortem on cows that died on the 17th, and found them to have had typical cases of splenic fever. I then separated the sick from the well.

The following is the symptoms and treatment of this herd:

RECORD.

| | | | | |
|---|---|---|---|---|
| July 18th, | 10.30 A.M., | No. 1, | Tem. | 107, very restless all night. |
| " | " | " 2, | " | 108. |
| " | " | " 3, | " | 106. |
| " | " | " 4, | " | 103½, restless, died at 5 P.M. |
| " | " | " 5, | " | 107½. |
| " | " | " 6, | " | 101¾, sinking, died at 7 P.M. |
| " | " | " 7, | " | 105¾, breathing very hard. |
| " | " | " 8, | " | 108. |
| " | " | " 9, | " | 104½. |

The treatment in all these cases was Fld. Ext. Eucalyptus Glob., ½ oz. doses, and Arsenicum Album, 1–100, 2 dr. doses, alternating every two hours.

| | | | | |
|---|---|---|---|---|
| July 19th, | 10.30 A.M., | No. 1, | Tem. | 104¾, restless all night. |
| " | " | " 2, | " | 106½. |
| " | " | " 3, | " | 103. |
| " | " | " 5, | " | 106⅓. |
| " | " | " 7, | " | 102, breathing almost normal. |
| " | " | " 8, | " | 105. |
| " | " | " 9, | " | 101⅓. |

The same treatment continued, they all ate, drank and ruminated some.

| | | | | |
|---|---|---|---|---|
| July 20th, | 12 M., | No. 1, | Tem. | 101, very little restlessness. |
| " | " | " 2, | " | 102½, very drowsy and stiff. |
| " | " | " 3, | " | 101. |
| " | " | " 5, | " | 102⅓. |
| " | " | " 7, | " | 101¼, breathing normal. |
| " | " | " 8, | " | 100¾. |
| " | " | " 9, | " | 101. |

Continued same treatment every three hours.

July 21st, 3.30 P.M. Temperature of all the cows was normal. They all ate and drank well, and had a fair flow of milk. No medicine.

On July 25th, I inoculated 9 cows and 1 calf with Chauveau's virus, received from Professor Liautard. The following is the method of vaccination: Clip the hair on inside of thin part of the ear. Make a small incision with a sharp-pointed bistoury. Clean off the small quantity of blood, and inject with hypodermic syringe two drops of the virus.

On July 26th, cow No. 3 had a very alarming high temperature: at 11.30 A.M., temperature 107, and at 7 P.M., temperature 108. All the rest of the cows had a slight rise in temperature. On July 27th, cow No. 3's temperature was 102½, and to all appearances well.

The ears of all the cows, where the inoculation was made, swelled to more or less extent.

The following is a history of the herd of cattle owned by the Hudson River State Hospital. On the 27th of March, 1887, I was called to make post-mortem examination on a young cow which had died the night previous. Results: On removing skin, the tissues appeared to be filled with dark hemorrhagic spots; the spleen very much enlarged, and of a very dark color; the abdomen contained a quantity of dark serum; the kidneys were dark and ecchymosed. The statement was made by Dr. Cleveland that they had lost from 10 to 12 head every year, at different periods, in the same manner for a number of years.

I proposed the vaccination of the entire herd with Chauveau's virus, to which the doctors of the hospital agreed, and on the 31st day of March I vaccinated the entire herd of 44 head.

See the appended table of temperatures after vaccination.

Of those vaccinated, two died after a lapse of four years and four months of splenic apoplexy, fortunately in a place where no cattle have been kept since.

I also vaccinated the herd of Mrs. Wiley, consisting of 23 head, and Mr. J. Rose's herd of 16 head. There was no table of temperatures kept of these two herds.

Since 1887, there has been but two cases of splenic apoplexy (the two of the Hudson River State Hospital) in the whole district.

Every year for over thirty years this disease has claimed its victims.

This is certainly very gratifying for the use of prophylactic treatment of this disease.

| No. | Temperature Before Injection. | Temperature After Injection. | | | | | |
|---|---|---|---|---|---|---|---|
| | March 31. | April 1. | Aplil 2. | April 3. | April 4. | April 5. | |
| 1 | 102 | 102 | 103 | 102 | 101 1-2 | 101 | |
| 2 | 100 | 102 1-4 | 102 | 101 1-2 | 102 | 100 3-4 | |
| 3 | 100 | 101 1-2 | 103 1-4 | 102 | 101 1-2 | 101 | |
| 4 | 100 1-2 | 101 | 102 | 102 | 101 | 100 1-2 | |
| 5 | 100 1-2 | 105 1-2 | 104 | 103 | 102 1-2 | 100 | |
| 6 | 100 3-4 | 102 1-4 | 103 | 104 1-2 | 102 1-2 | 101 | |
| 7 | 100 | 102 | 102 3-4 | 104 1-2 | 105 | 101 3-4 | |
| 8 | 100 | 101 | 101 | 101 | 102 | 101 | |
| 9 | 100 | 101 | 102 1-2 | 101 1-2 | 101 | 101 | |
| 10 | 100 | 101 1-4 | 102 1-2 | 102 | 100 3-4 | 100 | |
| 11 | 99 1-2 | 100 3-4 | 102 | 101 1-4 | 101 | 101 | |
| 12 | 100 | 101 1-2 | 103 1-4 | 103 1-2 | 102 | 101 1-2 | |
| 13 | 100 1-2 | 102 | 102 | 101 1-4 | 101 1-4 | 101 | |
| 14 | 100 | 100 3-4 | 102 | 101 | 101 | 100 1-2 | |
| 15 | 100 1-4 | 102 1-4 | 104 | 103 | 101 1-2 | 101 | |
| 16 | 101 | 102 | 102 1-2 | 101 | 101 | 101 1-2 | |
| 17 | 100 1-2 | 102 | 104 | 100 3-4 | 100 1-4 | 100 | |
| 18 | 100 3-4 | 102 | 104 3-4 | 101 1-4 | 101 | 101 | |
| 19 | 100 | 101 3-4 | 105 | 107 | 104 1-2 | 101 | Ear swelled bad. |
| 20 | 101 | 102 | 102 1-4 | 101 1-4 | 99 3-4 | 100 | |
| 21 | 100 | 101 3-4 | 102 1-2 | 100 1-4 | 100 3-4 | 100 | |
| 22 | 101 | 103 | 104 | 103 | 101 | 101 | |
| 23 | 100 1-4 | 102 | 102 1-2 | 99 1-2 | 100 1-2 | 100 | |
| 24 | 100 | 100 1-4 | 102 | 99 1-4 | 105 chill | 101 | |
| 25 | 100 | 102 1-2 | 103 | 100 1-4 | 100 | 100 1-2 | |
| 26 | 100 1-2 | 102 1-4 | 103 | 101 1-4 | 100 1-2 | 100 | |
| 27 | 101 | 103 3-4 | 103 1-4 | 103 | 100 1-4 | 101 | |
| 28 | 100 1-2 | 101 | 102 | 101 1-4 | 101 | 101 | |
| 29 | 99 1-4 | 101 1-2 | 102 | 100 | 101 | 100 1-2 | |
| 30 | 100 | 101 | 102 | 101 1-2 | 99 1-2 | 100 | |
| 31 | 100 | 101 1-2 | 102 | 101 1-4 | 101 1-4 | 101 | |
| 32 | 100 1-2 | 102 1-4 | 102 | 100 | 101 | 100 1-2 | |
| 33 | 100 | 103 3-4 | 102 | 103 | 102 1-2 | 100 3-4 | |
| 34 | 101 1-2 | 103 | 100 3-4 | 102 | 103 | 101 | |
| 35 | 100 1-4 | 102 3-4 | 102 | 101 | 100 | 100 1 4 | |
| 36 | 100 | 102 1-2 | 103 | 100 1-2 | 100 1-2 | 100 1-2 | |
| 37 | 100 3-4 | 103 | 102 | 101 | 100 | 100 1-2 | |
| 38 | 101 | 102 1-2 | 104 | 102 1-2 | 101 | 100 | |
| 39 | 101 1-4 | 103 | 102 1-2 | 101 | 101 1-2 | 101 | |
| 40 | 100 1-2 | 103 | 101 3-4 | 100 | 101 | 100 1-2 | |
| 41 | 101 1-2 | 104 | 102 | 101 | 100 1-2 | 100 3-4 | |
| 42 | 101 | 103 | 102 1-2 | 101 3-4 | 100 3-4 | 101 | |
| 43 | 100 | 103 1-2 | 105 | 103 | 101 1-2 | 100 1-4 | |
| 44 | 100 1-2 | 102 | 104 | 102 | 100 3-4 | 100 1-2 | |

